# Midfoot reconstruction with serratus anterior–rib osteomuscular free flap following oncological resection of synovial sarcoma

**DOI:** 10.1007/s10195-015-0341-3

**Published:** 2015-04-03

**Authors:** Bruno Battiston, Stefano Artiaco, Raimondo Piana, Elena Boux, Pierluigi Tos

**Affiliations:** 1 III Orthopaedic Division, Department of Orthopaedics and Traumatology, Orthopaedic and Trauma Center, Turin, Italy; 2 IV Orthopaedic Division, Department of Orthopaedics and Traumatology, Orthopaedic and Trauma Center, Via Zuretti 29, 10126 Turin, Italy; 3 Oncologic Orthopaedic Division, Department of Orthopaedics and Traumatology, Orthopaedic and Trauma Center, Turin, Italy; 4 Microsurgery Unit, Department of Orthopaedics and Traumatology, Orthopaedic and Trauma Center, Turin, Italy

**Keywords:** Microsurgery, Serratus anterior rib composite flap, Foot sarcoma

## Abstract

During recent decades, the concept of surgical treatment of malignant bone and soft tissue sarcomas has evolved, with the aim of preserving limb function. In this paper we report a case of metatarsal reconstruction by means of serratus and rib free flap after excision of a synovial sarcoma located in the dorsal aspect of the midfoot. Five years after the operation, the patient was free from recurrence and recovered full foot function. Amputation has been widely used in the past and this procedure still remains a valuable option when limb salvage is not possible. Nevertheless, in selected cases, reconstruction by means of composite free flaps may be successfully used for limb preservation in the treatment of malignant foot tumors after surgical excision.

## Introduction

During recent decades the concept of surgical treatment of malignant bone and soft tissue sarcomas has progressively evolved, and the preservation of the uninvolved parts of the extremities and the achievement of an acceptable limb function have become a major goal of oncological and reconstructive surgery [1].

The foot is a very difficult site for limb salvage surgery because bone, tendons and neurovascular structures are present in close proximity. Anatomical compartments may be therefore difficult to isolate and preserve during oncological excision. Furthermore, this area does not offer opportunities for performing local flaps effective for covering and reconstructing complex tissue defects.

Amputation has been widely used in the past and this procedure still remains a valuable option in order to eradicate the tumor and preserve an overall good prognosis for the patient in many cases. However, free flaps have now become a possible solution for limb salvage and functional preservation.

Until now the use of free flaps for the treatment of complex defects of the foot, secondary to oncological excision of bone and soft tissue tumors, was limited to a small number of patients. It has been described in the literature by a few authors, who reported isolated clinical cases or small clinical series [1–4].

In this paper we report a case of metatarsal reconstruction by means of free serratus and rib flap after excision of a synovial sarcoma located in the dorsal aspect of the midfoot. This flap was previously used in a few cases reported in the literature for traumatic defect of the lower extremities [5–8]. To our knowledge, this is the first case in which this kind of flap has been used for the functional reconstruction of the foot in a patient affected by a malignant disease.

## Case report

### Clinical history

**Fig. 1:**
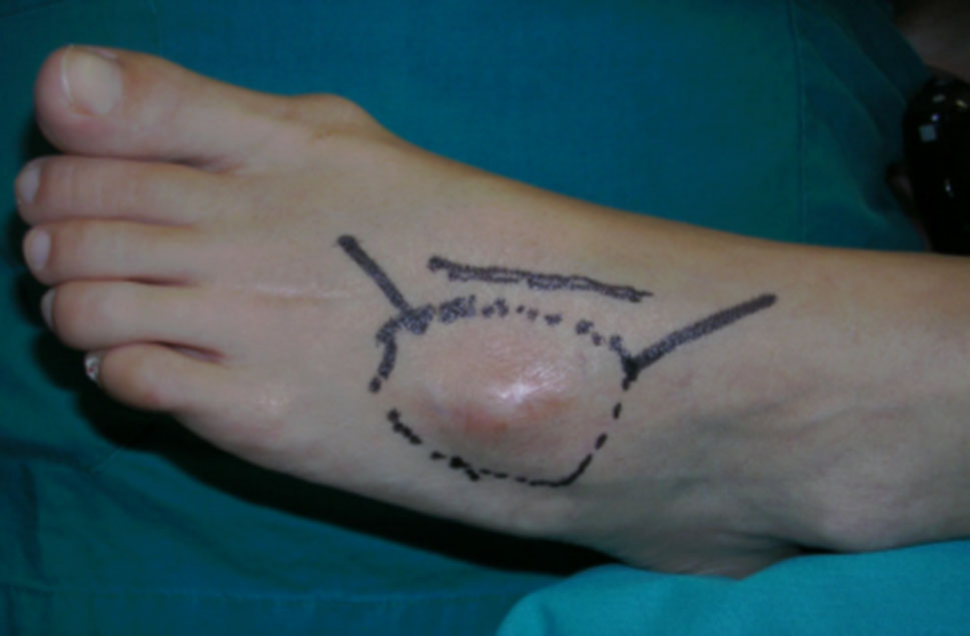
Preoperative clinical view

A 42-year-old woman came to our attention after two previous operations performed in another hospital for excision of a tumor mass located in the dorsal aspect of the midfoot. The patient presented 1 year after the second excisional biopsy which reported a diagnosis of neurinoma because of the recurrence of the tumor that was clinically located on the dorsal aspect of the foot in the 3rd metatarsal space (Fig. 1). No bone resection was performed during prior surgery. Magnetic resonance scanning confirmed the recurrence and defined the tumor extent, which did not involve neurovascular structures. Except for oncological disease, the patient was healthy and her clinical history was unremarkable. The specimens obtained during previous surgery were re-examined by a pathologist specialized in musculo-skeletal oncology, revealing a synovial sarcoma. The patient was then informed about the diagnosis and further surgical procedure was discussed in a consultation with oncological, orthopaedic, microsurgical and reconstructive staff. The operation was planned according to an oncological resection of the sarcoma including the 3rd and 4th metatarsal bones followed by reconstruction with osteomuscular serratus anterior and rib free flap in order to fill the defect and restore bone continuity for functional preservation. This flap was preferred to a vascularized fibular flap in order to avoid donor site morbidity because of the characteristics of the patient (highly active woman practising climbing and running).

**Fig. 2:**
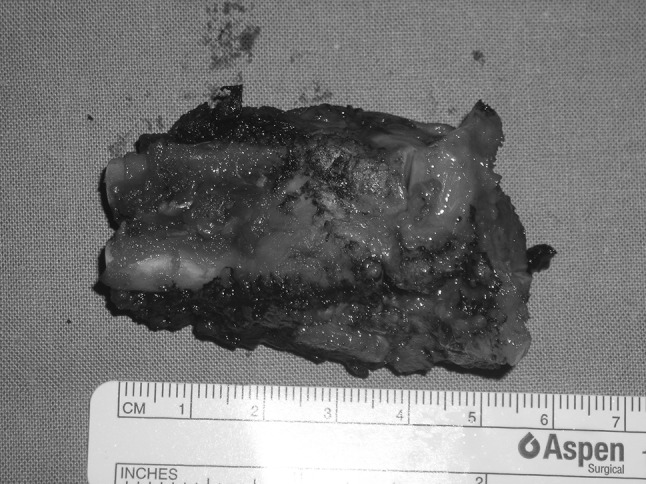
En-bloc resected tumor

**Fig. 3:**
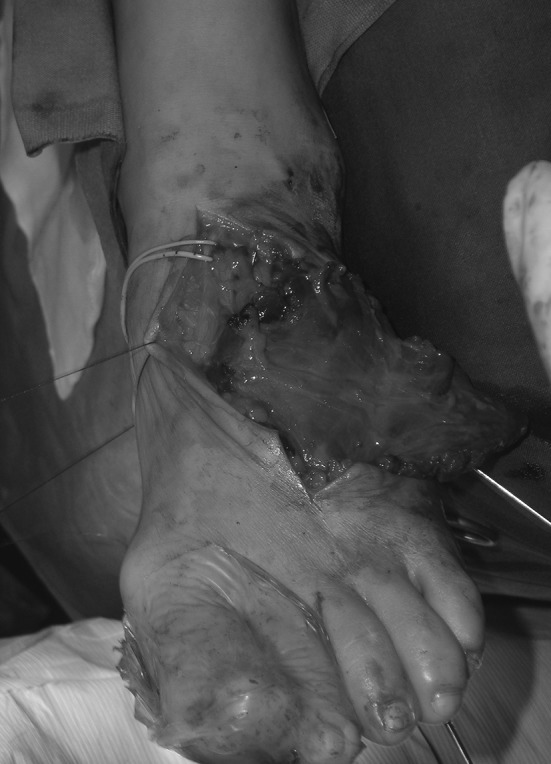
Serratus anterior–rib composite flap transferred and fixed on the foot

**Fig. 4:**
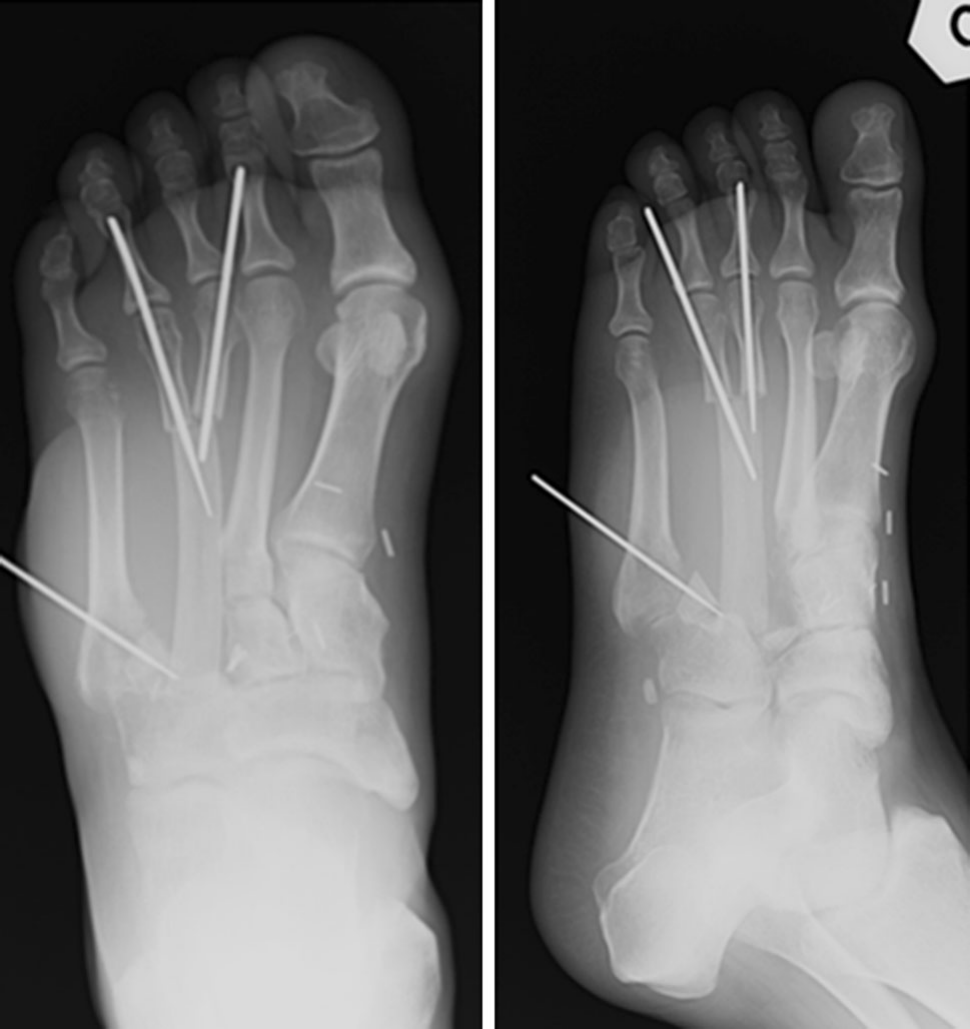
Postoperative radiographic oblique and antero-posterior view

En bloc tumor resection with wide margins, including the proximal and medial thirds of the 3rd and 4th metatarsal bone, was performed (Fig. 2). The plantar surface was disease-free, so the sensitivity of the foot was preserved. After tumor excision, a serratus anterior osteomuscular free flap including the 7th rib was transferred by means of termino-lateral suture to the dorsalis pedis artery and vein (Fig. 3). The rib was fixed to the distal third of the 3rd and 4th metatarsal bones and to the base of the fifth metatarsal bone by means of K-wires (Fig. 4). Skin grafting was used to cover the flap and a temporary short leg splint was applied. The specimen was sent to the pathologist for histological examination. The tumor had a diameter of 1 cm and was located in the central part of the specimen with wide resection margins. Macroscopically, bone and surrounding soft tissues were not infiltrated. Microscopically, the tumor was immune-reactive to vimentin. The final histological diagnosis confirmed the preoperative suspicion of biphasic synovial sarcoma.

### Postoperative course

**Fig. 5:**
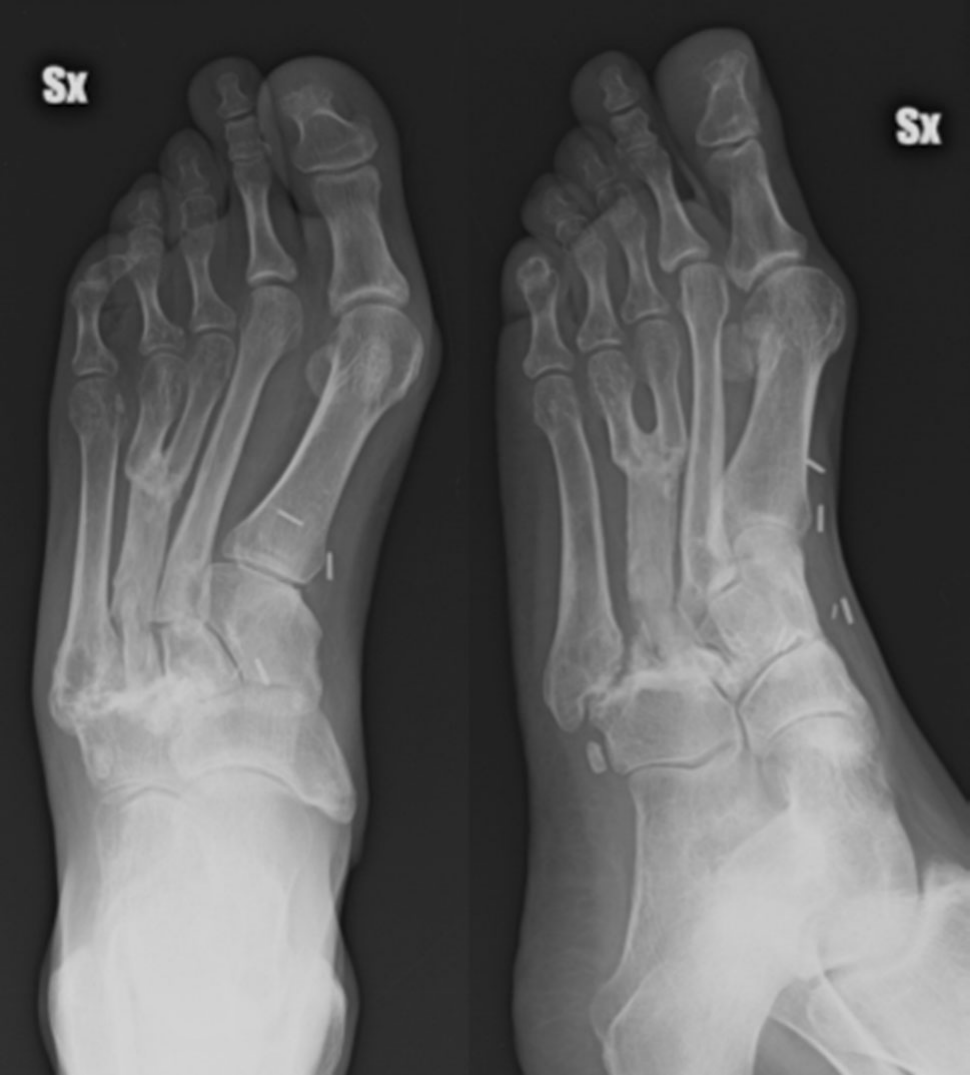
Radiographic oblique and antero-posterior view at follow-up

**Fig. 6:**
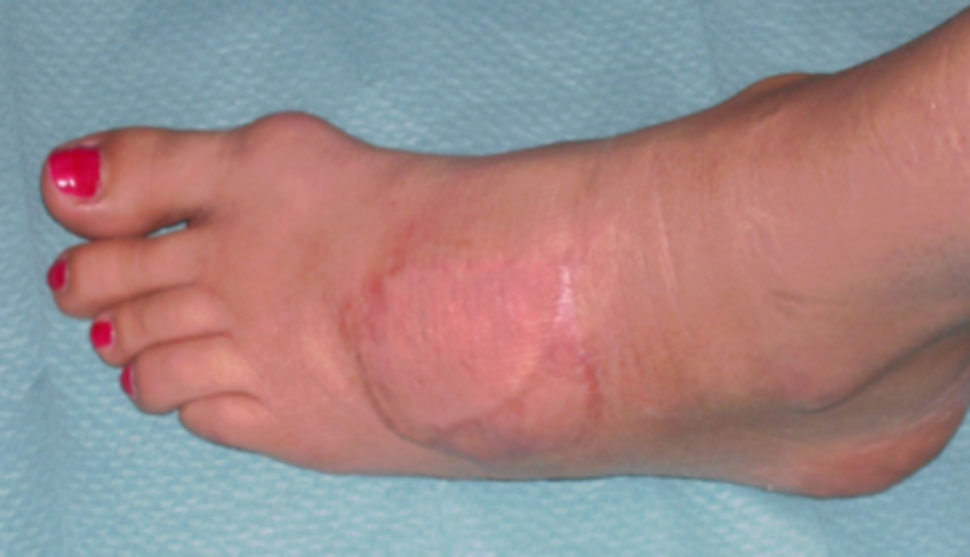
Clinical view at follow-up

The postoperative course was regular. K-wires were removed 2 months after the procedure. The vascularized rib graft healed with metatarsal bones and a progressive remodeling of the bones in the site of conjunction of the distal aspect of the transferred rib and proximal aspect of the 3rd and 4th metatarsal bones was observed (Fig. 5). No adjuvant therapy was performed. No donor site morbidity was observed due to rib resection. Partial and then full weight bearing were allowed in 2 and 3 months, respectively. Radiological healing was observed in 3 months. The patient returned to activities of daily life without limitation in 5 months and to sports activity (running and climbing) in 6 months. She did not show limitation of ankle or foot range of motion. Ambulation was physiological without limp and distance limitation. She had a periodical follow-up every 6 months, with clinical examination and magnetic resonance scan which did not show tumor recurrence. At last follow-up, 5 years after tumor excision and reconstructive procedure, the patient was free of recurrence and full functional recovery of the foot was observed (Fig. 6).

## Discussion

Serratus anterior and rib free flap based on the serratus branches of the thoracodorsal artery was described by Richards et al. [9]. Anatomical studies demonstrated that the serratus muscle has a consistent anatomy, a long pedicle and an excellent malleability, and that the anastomosis around the rib between its vessels and those arising from the intercostal artery allows harvesting of composite muscle and rib flap useful for covering complex defects [10].

Metatarsal and soft tissue reconstruction with serratus anterior and rib flap has been rarely described in sequelae of traumatic injuries of the foot. Kurokawa repaired four metatarsal bones in the same foot, transferring a flap which included the 5th and 7th ribs into the flap and Kitsiou used the serratus anterior and rib flap in three further cases without complications, achieving positive clinical results [6–8].

In our case we performed the reconstruction after excision of synovial sarcoma involving the dorsal aspect of the midfoot. To our knowledge, this is the first case in which the serratus anterior and rib free flap was used for the reconstruction of defect following excision of foot sarcoma.

As reported by Toma et al. [1], limb salvage surgery may be an alternative to amputation in the treatment of malignant bone and soft tissues tumors involving the extremities but it is not an option for all malignant tumors of the foot. Optimal reconstruction requires resistance to mechanical stresses and intact sensation of the plantar surface which is essential for foot function and skin integrity [1]. Thus, this surgery is a valuable option in the case of tumors with dorsal extension, as observed in our case. Metatarsal reconstruction is particularly important when the first and second metatarsal rays are involved by the sarcoma. In these cases, weight bearing and foot propulsion on the first and second metatarsal ray should be preserved in order to avoid postoperative sequelae including painful overuse syndrome, transfer metatarsalgia and osteoarthritis of tarsal–metatarsal joints. If reconstruction of the foot after wide resection does not allow for functionality, or requires continuous medical care, sparing surgery of the foot would be burdensome for the patient. In these cases amputation followed by prosthetic fitting would be the optimal choice of treatment.
